# Mortality in Critically Ill Patients with Liberal Versus Restrictive Transfusion Thresholds: A Systematic Review and Meta-Analysis of Randomized Controlled Trials with Trial Sequential Analysis

**DOI:** 10.3390/jcm14062049

**Published:** 2025-03-18

**Authors:** Daniel Arturo Jiménez Franco, Camilo Andrés Pérez Velásquez, David Rene Rodríguez Lima

**Affiliations:** 1 Escuela de Medicina y Ciencias de la Salud, Universidad del Rosario, Bogotá 111221, Colombia; 2 Critical and Intensive Care Medicine, Hospital Universitario Mayor-Mederi, Bogotá 111411, Colombia; 3 Grupo de Investigación Clínica, Escuela de Medicina y Ciencias de la Salud, Universidad del Rosario, Bogotá 111711, Colombia

**Keywords:** critical care, blood transfusion, restrictive transfusion, liberal transfusion, transfusion threshold

## Abstract

**Background/Objectives**: Anemia is common in critically ill patients, yet red blood cell (RBC) transfusion without active bleeding does not consistently improve outcomes and carries risks such as pulmonary injury, fluid overload, and increased costs. Optimal transfusion thresholds remain debated, with some guidelines recommending a restrictive target of 7 g/dL instead of a more liberal target of 9 g/dL. **Methods**: We conducted a systematic review and meta-analysis following PRISMA guidelines, searching PubMed, EMBASE, and LILACS from January 1995 to October 2024. Thirteen randomized controlled trials involving 13,705 critically ill adults were included, with 6855 assigned to liberal and 6850 to restrictive transfusion strategies. The risk of bias was assessed using the Cochrane Risk of Bias Tool 2, and the pooled effect sizes were estimated with a random-effects model. We registered the protocol in PROSPERO International Prospective Register of Systematic Reviews (CDR42024589225). **Results**: No statistically significant difference was observed in 30-day mortality between restrictive and liberal strategies (odds ratio [OR] 1.02; 95% confidence interval [CI], 0.83–1.25; I^2^ = 49%). Similarly, no significant differences emerged for the 90-day or 180-day mortality, hospital or intensive care unit (ICU) length of stay, dialysis requirement, or incidence of acute respiratory distress syndrome (ARDS). However, patients in the restrictive group received significantly fewer RBC units. The trial sequential analysis (TSA) indicated that the evidence accrued was insufficient to definitively confirm or exclude an effect on the 30-day mortality, as the required sample size was not reached. **Conclusions**: In conclusion, while our meta-analysis found no statistically significant difference in the short-term mortality between restrictive and liberal transfusion strategies, larger trials are needed to fully determine whether any clinically meaningful difference exists in critically ill populations.

## 1. Introduction

Anemia is a common problem among critically ill patients and is associated with adverse outcomes [1]. The primary rationale for correcting anemia in this population is to enhance oxygen delivery to tissues and mitigate potential hypoxic injury by increasing the oxygen-carrying capacity [2]. Although anemia has been associated with a higher mortality risk in critically ill patients [3], the practice of administering red blood cell (RBC) transfusions—particularly in the absence of active hemorrhage—has not consistently demonstrated unambiguous clinical benefit [4]. Furthermore, evidence increasingly indicates that RBC transfusions introduce significant risks, including acute transfusional reactions like transfusion-related acute lung injury, volume overload, infectious complications, and higher healthcare costs [5,6].

Transfusion indications and targets for critically ill patients remain controversial [7]. Currently, thresholds for restrictive and liberal transfusion strategies are defined primarily by absolute hemoglobin values, with limited consideration of additional triggers such as systemic perfusion, the impact of anemia, and oxygenation parameters. Moreover, there is considerable heterogeneity in the hemoglobin cut-off used to distinguish restrictive from liberal transfusion strategies. Recent guidelines from the European Society of Intensive Care Medicine (ESICM) recommend a restrictive transfusion threshold (hemoglobin 7 g/dL) over a liberal threshold (hemoglobin 9 g/dL), based on the moderate certainty of evidence and the absence of reported harms in most clinical contexts when employing a restrictive strategy [8]. Nevertheless, the randomized clinical trials supporting these recommendations have methodological limitations that may affect the confidence in the findings, owing to the imprecision or inconsistency across studies [9].

These uncertainties have contributed to considerable variability in transfusion practices, particularly in subpopulations with heightened susceptibility to complications associated with anemia, such as patients with acute myocardial infarction, those undergoing mechanical ventilation weaning, individuals with septic shock, or those with acute brain injury [7,10]. Although several recent investigations have attempted to clarify the optimal transfusion threshold, persistent questions remain, underscoring the importance of more definitive, large-scale data in guiding clinical decisions.

Consequently, we conducted a systematic review and meta-analysis encompassing a general intensive care unit (ICU) population, supplemented by a trial sequential analysis (TSA). The objective was to ascertain whether the observed effect of restrictive versus liberal transfusion thresholds on mortality is sufficiently precise to remain unchanged with further research. By evaluating whether the cumulative evidence meets the required sample size and significance boundaries, the TSA helps to determine if future studies are likely to alter the current conclusions. Ultimately, this approach provides a clearer perspective on whether restrictive transfusion strategies can be confidently recommended for critically ill patients in a broad range of clinical scenarios.

## 2. Materials and Methods

### 2.1. Protocol

This meta-analysis adhered to the guidelines outlined in the Preferred Reporting Items for Systematic Reviews and Meta-Analyses (PRISMA) [11]. The protocol was registered in International Prospective Register of Systematic Reviews (PROSPERO) in December 2024 (CDR42024589225).

### 2.2. Search Strategy and Data Extraction

A comprehensive search strategy was implemented via the PubMed, EMBASE, and Latin American and Caribbean Health Sciences Literature (LILACS) databases, covering the period from January 1995 to October 2024. Data extraction and eligibility assessment were standardized and carried out independently by two reviewers (J.F.D.A. and R.L.D.R.). In case of any disagreements, a third evaluator (P.V.C.A.) was included. Titles and abstracts were independently screened in duplicate using the Rayyan tool [12], and full-text versions of the relevant studies were subsequently retrieved. No language restrictions were applied. The complete search strategy, including the terms used, is detailed in the protocol registered in PROSPERO (registration number 589225).

### 2.3. Study Selection and Inclusion Criteria

The selection and inclusion of studies was based on the PICOS strategy, as described below:

- Patients and Setting: We included studies with adult critical care patients diagnosed with anemia. Patients under 18 years of age, pregnant individuals, or those with limitations of therapeutic effort were excluded.
- Interventions: Restrictive transfusion of RBCs with a target hemoglobin between 7 and 9 g/dL.
- Comparison: Liberal transfusion of RBCs with a target hemoglobin greater than 9 g/dL.
- Outcomes: Studies that evaluated mortality at 28 to 30 days as the primary outcome were included. If multiple data points were available within a study, all of the relevant data were considered.
- Study Type: Only randomized clinical trials were included. Exclusions were made for studies with standardized transfusion protocols, such as preoperative optimization in major surgery, orthopedic surgery, or cardiovascular surgery.

### 2.4. Study Selection and Data Collection Process

For studies meeting the inclusion criteria, information was compiled into individual extraction sheets to facilitate comparison. Any disagreements regarding inclusion, study quality, data adequacy, or the final classification of included studies were resolved through author consensus.

### 2.5. Data Items

Primary data extraction was conducted independently by two evaluators, who recorded their findings using standardized reporting forms (J.F.D.A. and R.L.D.R.). In cases of disagreement, a third evaluator was consulted to achieve consensus (P.V.C.A.). Extracted data included the authors, title, year of publication, study population, inclusion and exclusion criteria, number of participants, restrictive transfusion goal, liberal transfusion goal, primary outcome, and secondary outcomes.

### 2.6. Risk of Bias in Individual Studies

The risk of bias assessment was performed using the Cochrane Risk of Bias Tool 2 (RoB 2), which focuses on the following five domains: randomization, intervention, missing outcome data, outcome measurement, and outcome reporting [13]. Accordingly, the risk of bias was categorized individually for each outcome as low, high, or unclear. The grade of evidence assessment was also performed according to the recommendations of the Grading of Recommendations Assessment, Development, and Evaluation working group using the GRADEpro software (v.3.0.; McMaster University).

### 2.7. Statistical Analysis

#### 2.7.1. Analysis of Individual Studies and Summary Measures

The meta-analysis was conducted using the R statistical package (version 4.3.1) and the DerSimonian–Laird random-effects model. For binary outcomes, odds ratios were estimated to evaluate the association between categorical variables of interest, and the Mantel–Haenszel method was employed to determine the study weights. Continuous outcomes were assessed using the Hedges method, with the results reported as mean differences. A 95% prediction interval was also provided for the primary analysis. Statistical significance was set at *p* < 0.05.

Heterogeneity was assessed using the Chi^2^ test, with *p* > 0.01 indicating consistency across studies. In addition, Higgins’ I^2^ index was calculated to quantify the proportion of variability in effect sizes not attributable to chance. Heterogeneity was categorized as follows: I^2^ = 0–24.9% (none), I^2^ = 25.0–49.9% (low), I^2^ = 50.0–74.9% (moderate), and I^2^ > 75% (high).

#### 2.7.2. Analysis of Risk of Bias Across Studies

Publication bias was assessed graphically using the funnel plot and Egger’s linear regression.

#### 2.7.3. Subgroup Analysis and Trial Sequential Analysis

The robustness of the primary results was evaluated using TSA conducted in R Studio (v.4.3.1) with the RTSA statistical package. The estimated effect was assessed using the OR obtained from the forest plot, and the cumulative evidence was compared against trial sequential monitoring boundaries. For this analysis, a random-effects model was employed, assuming an 80% power, a 5% type I error, and a clinical significance threshold defined by an OR of 0.80. By applying the TSA, we determined whether the accrued evidence reached the required information size to draw firm conclusions or if additional studies would be necessary or futile to confirm the observed effect. Additionally, a subgroup analysis was conducted according to the specific intensive care populations examined (medical ICU, sepsis, cardiovascular, burns, and acute brain injury) to evaluate the effect of a liberal transfusion strategy compared with a restrictive transfusion approach on 30-day mortality outcomes.

## 3. Results

### 3.1. Study Selection

**Figure 1:**
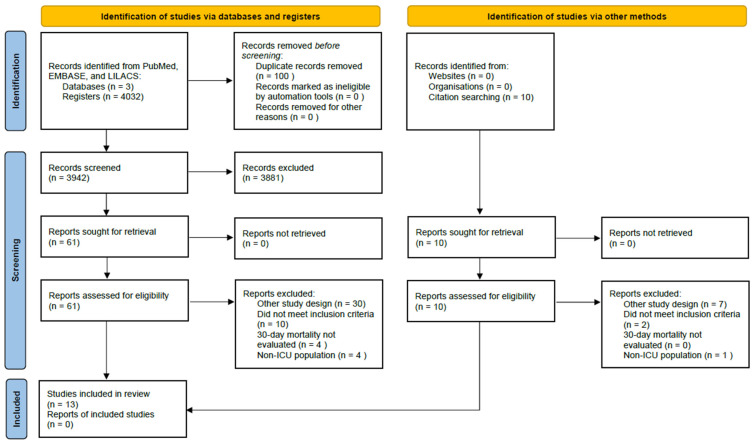
Flow chart of the study selection. LILACS: Latin American and Caribbean Health Sciences Literature. ICU: intensive care unit. EMBASE: Excerpta Medica Database. PubMed: Public MEDLINE National Library of Medicine United States [11].

The primary search identified 71 studies. A total of 54 articles were excluded due to duplicates or failure to meet the inclusion criteria. Additionally, three studies were excluded because the original database could not be acquired for data extraction. Finally, 13 randomized clinical trials were included for review and final analysis [14,15,16,17,18,19,20,21,22,23,24,25,26] (Figure 1).

### 3.2. Study Characteristics

A total of 13,705 patients were included in the meta-analysis, with 6855 in the liberal transfusion group and 6850 in the restrictive transfusion group. The general characteristics of the included studies are summarized in Table 1. Among the 13 studies, 5 were conducted in patients with cardiovascular conditions [16,18,21,23,25], 2 in those with septic shock [17,20], 3 in medical ICU patients [14,15,19], 1 in burn patients [22], and 2 in acute brain injury patients [24,26]. Of these, seven studies used a liberal hemoglobin target between 9 and 10 g/dL, while six studies used a target greater than 10 g/dL. The general characteristics of the included studies are summarized in Table 1.

### 3.3. Syntheses of Results

**Figure 2:**
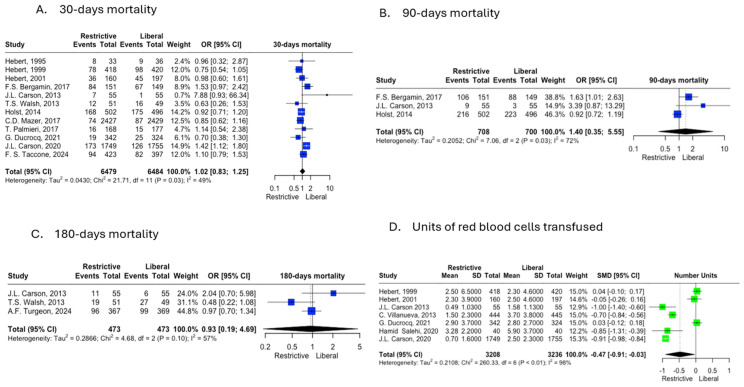
Forest plot for the main outcomes comparing restrictive and liberal transfusion strategies. (**A**). 30-day mortality. (**B**). 90-day mortality. (**C**). 180-day mortality. (**D**). Units of red blood cells (RBCs) transfused, green color indicates a statistically significant result [14,15,16,17,18,19,20,21,22,23,24,25,26].

The primary 30-day mortality outcome was reported by 12 studies, and the meta-analysis showed no significant difference between groups (OR 1.02; 95% CI 0.83–1.25; *p* = 0.03, I^2^ = 49%). The 90-day mortality outcome was reported by three studies, and this analysis also revealed no significant differences between groups (OR 1.40; 95% CI 0.35–5.55; *p* = 0.03, I^2^ = 72%). The 180-day mortality outcome was assessed by three studies, showing no significant differences between groups (OR 0.93; 95% CI 0.19–4.69; *p* = 0.10, I^2^ = 57%) (Figure 2).

Hospital length of stay was reported in four studies and showed no significant differences between groups (Standardized Mean Difference [SMD] −0.05; 95% CI −0.24 to 0.14; *p* = 0.05; I^2^ = 62%). Similarly, the ICU length of stay was reported by three studies and revealed no significant differences between groups (SMD −0.04; 95% CI −0.36 to 0.27; *p* = 0.12; I^2^ = 53%) (Supplementary Material Figure S1).

Among the secondary outcomes assessed, the risk of requiring dialysis was reported by four studies, demonstrating no significant differences between groups (OR 0.86; 95% CI 0.35–2.11; *p* = 0.04; I^2^ = 63%). The incidence of ARDS, reported by three studies, also showed no significant differences (OR 0.65; 95% CI 0.07–6.12; *p* = 0.05; I^2^ = 66%) (Supplementary Material Figure S1). Finally, the number of RBC units transfused was reported by seven studies and was significantly lower in the restrictive transfusion strategy group (SMD −0.47; 95% CI −0.91 to −0.03; *p* < 0.01; I^2^ = 98%) (Figure 2).

### 3.4. Risk of Bias

**Figure 3:**
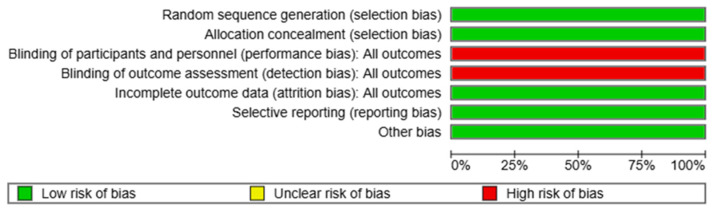
Distribution of the risk of bias assessment.

Risk of bias was assessed using the RoB-2 tool (Figure 3), showing that most studies were classified as having a low overall risk of bias. However, all of the studies were deemed to have a high risk of bias in the performance and detection bias domains. Consequently, a sensitivity analysis was conducted to evaluate the individual contribution of each study to the overall effect estimate. The grade of evidence is shown in Supplementary Material Figure S2.

### 3.5. Additional Analysis

#### 3.5.1. Sensitivity Analysis

A sensitivity analysis was performed to assess the individual contribution of each study to the overall effect estimate, and no single study demonstrated a statistically significant impact on the results (Supplementary Material Figure S3).

#### 3.5.2. Trial Sequential Analysis

**Figure 4:**
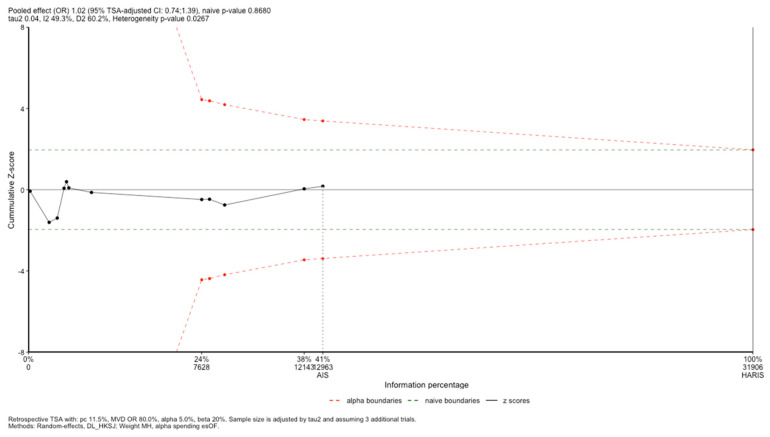
Trial sequential analysis. AIS: achieved information size; HARIS: heterogeneity-adjusted required information size for a non-sequential meta-analysis; MVD: mean value difference; DL_HKSJ: DerSimonian–Laird with Hartung–Knapp–Sidik–Jonkman adjustment; esOF: Lan and DeMets version of O’Brien–Fleming boundaries; TSA: trial sequential analysis.

The TSA was performed to assess the robustness of the findings for the 30-day mortality outcome, using an OR of 1.02 derived from the primary meta-analysis. This analysis indicated that the results were not robust, as the cumulative Z-score remained in the nonsignificant region (TSA-adjusted OR 0.74–1.39; *p* = 0.8680) (Figure 4). Moreover, the total sample size analyzed represented only 41% of the required 31,906 participants, which would be needed to achieve a 90% statistical power for detecting a significant effect on 30-day mortality.

#### 3.5.3. Subgroup Analysis

A subgroup analysis was performed according to the included populations—medical ICU, sepsis, and cardiovascular. The burns and acute brain injury subgroups were excluded, as only one study in each group assessed the 30-day mortality. In the subgroup analysis, no statistically significant difference in the risk of 30-day mortality was observed between the liberal and restrictive transfusion strategies (Supplementary Material Figure S4).

#### 3.5.4. Publication Bias

No evidence of publication bias was detected based on visual inspection of the funnel plot, which showed a symmetrical distribution of the points (Supplementary Material Figure S5).

## 4. Discussion

In this meta-analysis, the use of restrictive transfusion strategies (7–9 g/dL) compared to liberal strategies (>9 g/dL) in critically ill patients did not show a significant reduction in the 30-day mortality (OR 1.02; 95% CI 0.83–1.25; *p* = 0.03; I^2^ = 49%). After performing the TSA, the cumulative Z-score line remained in the zone of nonsignificance and did not reach the required sample size to either refute or confirm the hypothesis. Consequently, these results are not conclusive, and additional studies are necessary to determine the magnitude of the effect. Nevertheless, while additional large-scale studies are required to conclusively exclude any small yet potentially significant advantages of restrictive transfusion strategies, the current evidence indicates that, if such an effect exists, its magnitude is likely too modest to have a meaningful clinical impact. Overall, our findings highlight the absence of harm associated with using restrictive transfusion strategies and underscore the potential to reduce costs and adverse events associated with the increased number of RBC units required under a liberal transfusion strategy. Furthermore, these findings indicate that a restrictive transfusion strategy is both safer and more cost-effective than a liberal approach, and that employing a liberal transfusion strategy based on higher hemoglobin levels does not offer sufficient clinical benefit to justify its continued use.

In critically ill patients, hypoxia reflects an imbalance between oxygen delivery and consumption across various organ systems, often arising from a confluence of factors, such as impaired pulmonary function, hemodynamic instability, and reduced oxygen-carrying capacity [27]. Prolonged periods of hypoxia, commonly referred to as the “oxygen debt”, have been linked to adverse outcomes, including organ failure and elevated mortality [28,29]. Anemia frequently exacerbates this condition by directly diminishing hemoglobin’s ability to transport oxygen to vital tissues, especially in individuals with compromised cardiovascular reserve [30]. Restrictive transfusion strategies, which typically employ hemoglobin thresholds of 7–9 g/dL, are often viewed to minimize exposure to allogeneic red blood cells (RBCs). By contrast, liberal strategies set higher targets, generally above 9 g/dL, on the premise that an increased oxygen-carrying capacity could improve the clinical outcomes and mitigate organ hypoxia [3]. Despite this intuitive rationale, evidence from multiple randomized controlled trials (RCTs) has challenged the necessity of liberal thresholds in the absence of acute hemorrhage [4,5,6]. Nevertheless, it is crucial to recognize that oxygen delivery in critical illness is multifactorial. While liberal transfusions might bolster hemoglobin levels, other determinants, such as cardiac output, microcirculatory function, ventilation status, and the patient’s underlying pathology, ultimately govern tissue oxygenation [12]. As such, the hypothesized benefits of higher hemoglobin targets may not always translate into demonstrable improvements in survival or organ function.

Most studies assessing the impact of transfusion strategies on mortality risk have used a restrictive transfusion threshold (7–9 g/dL) as the intervention group, requiring the demonstration of either benefit or no harm compared with more liberal thresholds [31]. However, previous meta-analyses [8,9,32], including our own, have not identified an increased risk of adverse outcomes with restrictive strategies, suggesting that the mortality risk likely does not increase, and that other safety- and cost-related outcomes may potentially be improved.

The body of evidence on this topic has grown considerably over the past 50 years, albeit with inconclusive results [33]. Traditional meta-analyses can evaluate the pooled effect size of an intervention on a specific outcome, but they are limited in determining whether the sample size and statistical power are sufficient to accept or reject the study hypothesis [34]. TSA is a frequentist method that, based on accepted type I and type II error thresholds, assesses whether meta-analysis results are robust enough to remain unaffected by subsequent studies [35]. This approach allows for the confirmation or rejection of the pooled effect estimate and clarifies whether the required sample size has been reached to draw definitive conclusions [36].

In our study, we prespecified an OR of 0.80 as the threshold for clinical significance for 30-day mortality. Assuming an 80% power and a 5% type I error rate, it was not possible to demonstrate the absence of an effect due to an inadequate sample size. These findings suggest that, if there is any benefit in terms of reducing the 30-day mortality in the restrictive transfusion group, it is likely too small to be clinically meaningful, and thus does not justify further studies using the same methodological design to confirm it. This approach suggests that additional criteria, beyond relying solely on hemoglobin levels, should be incorporated and evaluated in clinical studies to demonstrate the impact of correcting anemic hypoxia in critically ill patients without active bleeding [30].

This meta-analysis has several limitations. First, although we included all of the clinical trials that compared restrictive and liberal transfusion strategies in critically ill patients, the total number of patients and events did not reach the required sample size. Second, the initial heterogeneity among the studies was moderate; however, the subgroup analyses did not demonstrate any differences in effect, and the sensitivity analyses indicated that no single study substantially contributed to this heterogeneity. Still, there is likely clinical heterogeneity given the inclusion of a general ICU population, which may limit the external validity of the results, particularly for populations vulnerable to hypoxia.

## 5. Conclusions

In conclusion, our meta-analysis did not demonstrate a statistically significant difference in the 30-day mortality when comparing restrictive versus liberal transfusion strategies in a general ICU population (OR 1.02; 95% CI 0.83–1.25; *p* = 0.03; I^2^ = 49%). Moreover, the TSA suggests that these findings are neither robust nor conclusive, indicating the need for additional randomized studies are required to provide convincing outcome data in the selected patient population to achieve sufficient statistical power.

## Figures and Tables

**Table 1:** General characteristics of the included studies. Data are presented as No. (%), No./Total No. (%), percentage, or mean (SD) unless otherwise indicated.

Study	Year	Sample Size	Age in Years, Mean ± SD, Median (IQR)	Sex, Male/Female	Population	Restrictive Thresholds	n=	Liberal Thresholds	n=	30-Day Mortality (Restrictive/Liberal) no (%).
Hébert et al. [14]	1995	69	58.6 ± 15	33/36	Medical ICU	7–9 g/dL	33	10–12 g/dL	36	8 (24%)/9 (25%)
Hébert et al. [15]	1999	838	57.1 ± 18.1	524/314	Medical ICU	7–9 g/dL	418	10–12 g/dL	420	78 (18.7%)/98 (23%)
Hebert et al. [16]	2001	357	64.0 ± 14.1	216/141	Cardiovascular	7–9 g/dL	160	10–12 g/dL	197	36 (23%)/45 (23%)
Bergamin et al. [17]	2017	300	61.6 ± 12.9	154/146	Septic shock	<7 g/dL	151	<9 g/dL	149	67 (45%)/84 (55.6%)
Carson et al. [18]	2013	110	70.8 ± 12.8	55/55	Cardiovascular	<8 g/dL	55	<10 g/dL	55	7 (13%)/1 (1.8%)
Walsh et al. [19]	2013	100	67 ± 7	60/40	Medical ICU	7–9 g/dL	51	9–11 g/dL	49	12 (23.5%)/16 (32.7%)
Holst et al. [20]	2014	998	67 (57–73)	531/467	Septic shock	≤7 g/dL	502	<9 g/dL	436	168 (31.6%)/175 (34.8%)
Mazer et al. [21]	2017	4856	72 ± 10	3139/1717	Cardiovascular	≤7.5 g/dL	2427	≤9.5 g/dL	2429	74 (3%)/87 (3.6%)
Palmieri et al. [22]	2017	345	41 (30–55)	273/72	Burns	7 g/dL	168	≥10 g/dL	177	16 (9.5%)/15 (8.5%)
Ducrocq et al. [23]	2021	666	78 (69–85)	201/184	Cardiovascular	8–10 g/dL	342	>11 g/dL	324	19 (5.6%)/25 (7.7%)
Turgeon et al. [24]	2024	736	48.9 ± 18.8	937/201	Acute brain injury	≤7 g/dL	371	<10 g/dL	371	NA
Carson et al. [25]	2020	3504	72.1 ± 11.6	1137/2367	Cardiovascular	7–8 g/dL	1749	9–10 g/dL	1755	173 (9.9%)/146 (8.3%)
Taccone et al. [26]	2024	820	52 ± 16	436/384	Acute brain injury	<7 g/dL	423	<9 g/dL	397	82 (20.7%)/94 (22.5%).

## Data Availability

Supporting reported results can be found in the Supplementary Materials.

## Supplementary Materials

The following supporting information can be downloaded at: https://www.mdpi.com/article/10.3390/jcm14062049/s1, Figure S1: Forest plots for A. renal replacement therapy, B. acute respiratory distress syndrome, C. ICU stay, and D. hospital stay; Figure S2: Grade of evidence assessment; Figure S3: Sensitivity analysis; Figure S4: Subgroup analysis 30-days mortality. Figure S5. Funnel Plot for 30-days mortality.

## Abbreviations

The following abbreviations are used in this manuscript: ARDS: acute respiratory distress syndrome; CI: confidence interval; ESICM: European Society of Intensive Care Medicine; GRADE: Grading of Recommendations Assessment, Development, and Evaluation software; ICU: intensive care unit; LILACS: Latin American and Caribbean Health Sciences Literature; OR: odds ratio; PICOS: Population, Intervention, Comparison, Outcomes, Study design; PRISMA: Preferred Reporting Items for Systematic Reviews and Meta-Analyses; PROSPERO: International Prospective Register of Systematic Reviews; RBC: red blood cell; RoB 2: Cochrane Risk of Bias Tool 2; SMD: standardized mean difference; TSA: trial sequential analysis.
