# Supplementary material for: Mortality in Critically Ill Patients with Liberal Versus Restrictive Transfusion Thresholds: A Systematic Review and Meta-Analysis of Randomized Controlled Trials with Trial Sequential Analysis
Source: J Clin Med. 2025 Mar 18;14(6):2049. doi: 10.3390/jcm14062049 (PMC11943106; doi:10.3390/jcm14062049)
Supplement: Supplementary file 1 [file jcm-14-02049-s001.zip › jcm-3527004-supplementary.pdf]

Mortality in Critically Ill Patients with Liberal versus Restrictive Transfusion Thresholds: A Systematic Review and Meta-analysis of Randomized Controlled Trials with Trial Sequential Analysis

Supplementary Material

Daniel Arturo Jiménez Franco[1,2]

Camilo Andrés Pérez Velásquez[1,2] (ORCID 0009-0002-6613-6456)

David Rene Rodriguez Lima[2,3] (ORCID 0000-0002-7089-018X)

1. Escuela de Medicina y Ciencias de la Salud, Universidad del Rosario, Bogotá, Colombia
2. Critical and Intensive Care Medicine, Hospital Universitario Mayor-Mederi, Bogotá, Colombia
3. Grupo de Investigación Clínica, Escuela de Medicina y Ciencias de la Salud, Universidad del Rosario, Bogotá, Colombia.

Corresponding author: Camilo Andrés Pérez Velásquez: perezcamilo35@gmail.com

A. Renal replacement therapy

Study	Restrictive Events	Restrictive Total	Liberal Events	Liberal Total	Weight	OR [95% CI]
Hebert, 1999	6	78	10	98	17.3%	0.73 [0.25; 2.11]
Hebert, 2001	13	160	12	197	23.3%	1.36 [0.60; 3.08]
F.S. Bergamin, 2017	18	151	13	149	25.1%	1.42 [0.67; 3.00]
F. S. Taccone, 2024	27	423	50	397	34.3%	0.47 [0.29; 0.77]
**Total (95% CI)**		**812**		**841**	**100.0%**	**0.86 [0.35; 2.11]**

Heterogeneity: $Tau^2 = 0.1694$; $Chi^2 = 8.21$, df = 3 (P = 0.04); $I^2 = 63\%$

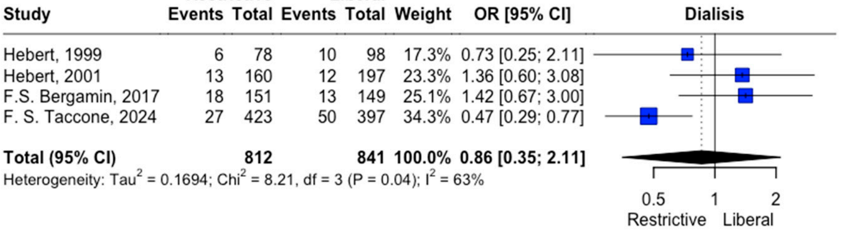

B. Acute respiratory distress syndrome

Study	Restrictive Events	Restrictive Total	Liberal Events	Liberal Total	Weight	OR [95% CI]
Hebert, 1999	32	418	48	420	42.5%	0.64 [0.40; 1.03]
G. Ducrocq, 2021	1	342	7	324	15.7%	0.13 [0.02; 1.09]
F. S. Taccone, 2024	36	423	29	397	41.8%	1.18 [0.71; 1.96]
**Total (95% CI)**		**1183**		**1141**	**100.0%**	**0.65 [0.07; 6.12]**

Heterogeneity: $Tau^2 = 0.5851$; $Chi^2 = 5.88$, df = 2 (P = 0.05); $I^2 = 66\%$

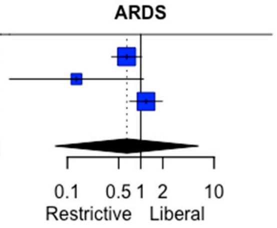

C. ICU stay

Study	Restrictive Mean	Restrictive SD	Restrictive Total	Liberal Mean	Liberal SD	Liberal Total	Weight	SMD [95% CI]
Hebert, 1999	11.00	10.7000	418	11.50	11.3000	420	38.7%	-0.05 [-0.18; 0.09]
Hebert, 2001	9.20	9.1000	160	11.30	11.6000	197	25.4%	-0.20 [-0.41; 0.01]
F. S. Taccone, 2024	22.50	15.6000	423	21.40	15.7000	297	35.9%	0.07 [-0.08; 0.22]
**Total (95% CI)**			**1001**			**914**	**100.0%**	**-0.04 [-0.36; 0.27]**

Heterogeneity: $Tau^2 = 0.0079$; $Chi^2 = 4.30$, df = 2 (P = 0.12); $I^2 = 53\%$

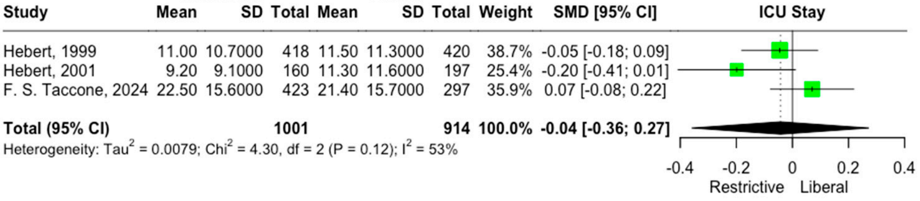

D. Hospital stay

Study	Restrictive Mean	Restrictive SD	Restrictive Total	Liberal Mean	Liberal SD	Liberal Total	Weight	SMD [95% CI]
Hebert, 1999	34.80	19.5000	418	35.50	19.4000	420	28.0%	-0.04 [-0.17; 0.10]
Hebert, 2001	33.00	19.6000	111	35.10	19.5000	147	15.7%	-0.11 [-0.35; 0.14]
C. Villanueva, 2013	9.60	8.7000	444	11.50	12.8000	445	28.5%	-0.17 [-0.31; -0.04]
F. S. Taccone, 2024	45.50	39.5000	423	42.00	34.8000	397	27.8%	0.09 [-0.04; 0.23]
**Total (95% CI)**			**1396**			**1409**	**100.0%**	**-0.05 [-0.24; 0.14]**

Heterogeneity: $Tau^2 = 0.0094$; $Chi^2 = 7.85$, df = 3 (P = 0.05); $I^2 = 62\%$

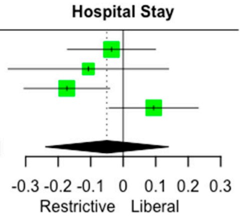

Figure S1. Forest plot for A. Renal replacement therapy. B. Acute respiratory distress syndrome. C. ICU stay. D. Hospital stay.

Summary of findings:

RESTRICTIVE TRANSFUSION STRATEGY compared to LIBERAL TRANFUSION STRATEGY for ANEMIA IN CRTICAL CARE

Patient or population: ANEMIA IN CRTICAL CARE

Setting:

Intervention: RESTRICTIVE TRANSFUSION STRATEGY

Comparison: LIBERAL TRANFUSION STRATEGY

Outcomes	Anticipated absolute effects* (95% CI)		Relative effect (95% CI)	№ of participants (studies)	Certainty of the evidence (GRADE)	Comments
	Risk with LIBERAL TRANFUSION STRATEGY	Risk with RESTRICTIVE TRANSFUSION STRATEGY				
30-DAYS MORTALITY (30-DAYS MORTALITY) follow-up: mean 30 days	115 per 1000	**117 per 1000** (97 to 140)	**OR 1.02** (0.83 to 1.25)	12963 (12 RCTs)	⨁⨁⨁⨁ High	
90-DAYS MORTALITY follow-up: 90 days	463 per 1000	**547 per 1000** (232 to 827)	**OR 1.40** (0.35 to 5.55)	1408 (3 RCTs)	⨁⨁⨁⨁ High	
180-DAYS MORTALITY follow-up: 180 days	279 per 1000	**265 per 1000** (69 to 645)	**OR 0.93** (0.19 to 4.69)	946 (3 RCTs)	⨁⨁⨁⨁ High	
RENAL REPLACEMENT THERAPY follow-up: 180 days	101 per 1000	**88 per 1000** (38 to 192)	**OR 0.86** (0.35 to 2.11)	1653 (4 RCTs)	⨁⨁⨁⨁ High	
ACUTE RESPIRATORY DISTRESS SYNDROME (ARDS) follow-up: 180 days	74 per 1000	**49 per 1000** (6 to 327)	**OR 0.65** (0.07 to 6.12)	2324 (3 RCTs)	⨁⨁⨁⨁ High	
UNITS OF RED BLOOD CELLS TRANSFUSED (RBC UNITS) follow-up: 30 days	7 per 1000	**-3 per 1000** (-6 to -0)	**SMD -0.47** (-0.91 to -0.03)	6444 (7 RCTs)	⨁⨁⨁⨁ High	

***The risk in the intervention group** (and its 95% confidence interval) is based on the assumed risk in the comparison group and the **relative effect** of the intervention (and its 95% CI).

CI: confidence interval; **OR:** odds ratio

GRADE Working Group grades of evidence
High certainty: we are very confident that the true effect lies close to that of the estimate of the effect.
Moderate certainty: we are moderately confident in the effect estimate: the true effect is likely to be close to the estimate of the effect, but there is a possibility that it is substantially different.
Low certainty: our confidence in the effect estimate is limited: the true effect may be substantially different from the estimate of the effect.
Very low certainty: we have very little confidence in the effect estimate: the true effect is likely to be substantially different from the estimate of effect.

Figure S2. Grade of evidence assessment.

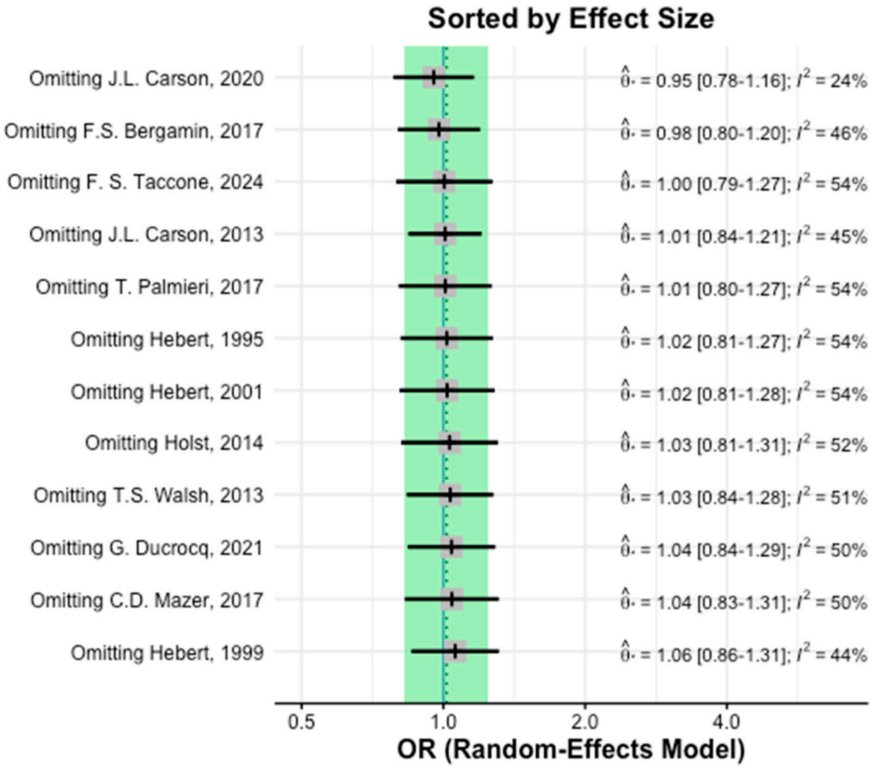

Figure S3. Sensitivity analysis.

B. Cardiovascular

Study	Restrictive Events	Restrictive Total	Liberal Events	Liberal Total	Weight	OR [95% CI]
Hebert, 2001	36	160	45	197	20.0%	0.98 [0.60; 1.61]
C.D. Mazer, 2017	74	2427	87	2429	29.9%	0.85 [0.62; 1.16]
G. Ducrocq, 2021	19	342	25	324	15.4%	0.70 [0.38; 1.30]
J.L. Carson, 2020	173	1749	126	1755	34.8%	1.42 [1.12; 1.80]
**Total (95% CI)**		**4678**		**4705**	**100.0%**	**1.01 [0.62; 1.65]**

Heterogeneity: $Tau^2 = 0.0520$; $Chi^2 = 9.21$, df = 3 (P = 0.03); $I^2 = 67\%$

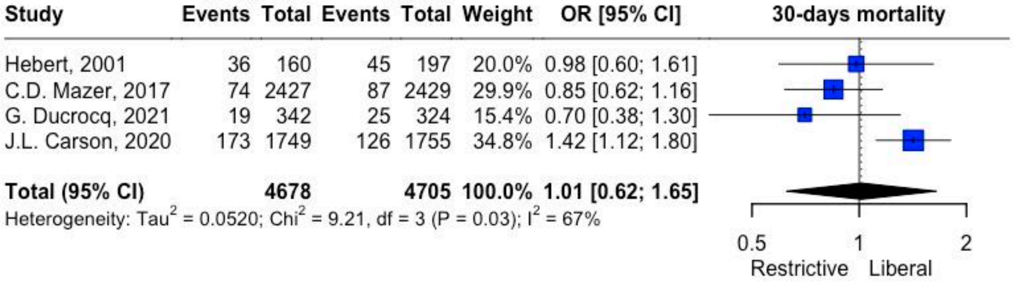

A. Septic shock

Study	Restrictive Events	Restrictive Total	Liberal Events	Liberal Total	Weight	OR [95% CI]
F.S. Bergamin, 2017	84	151	67	149	43.0%	1.53 [0.97; 2.42]
Holst, 2014	168	502	175	496	57.0%	0.92 [0.71; 1.20]
**Total (95% CI)**		**653**		**645**	**100.0%**	**1.15 [0.05; 28.17]**

Heterogeneity: $Tau^2 = 0.0935$; $Chi^2 = 3.61$, df = 1 (P = 0.06); $I^2 = 72\%$

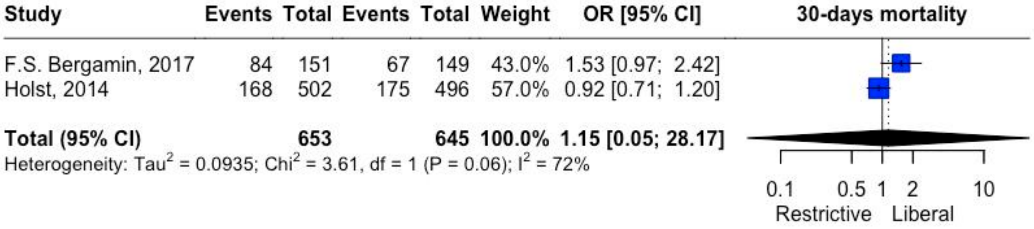

C. Medical ICU

Study	Restrictive Events	Restrictive Total	Liberal Events	Liberal Total	Weight	OR [95% CI]
F.S. Bergamin, 2017	84	151	67	149	43.0%	1.53 [0.97; 2.42]
Holst, 2014	168	502	175	496	57.0%	0.92 [0.71; 1.20]
**Total (95% CI)**		**653**		**645**	**100.0%**	**1.15 [0.05; 28.17]**

Heterogeneity: $Tau^2 = 0.0935$; $Chi^2 = 3.61$, df = 1 (P = 0.06); $I^2 = 72\%$

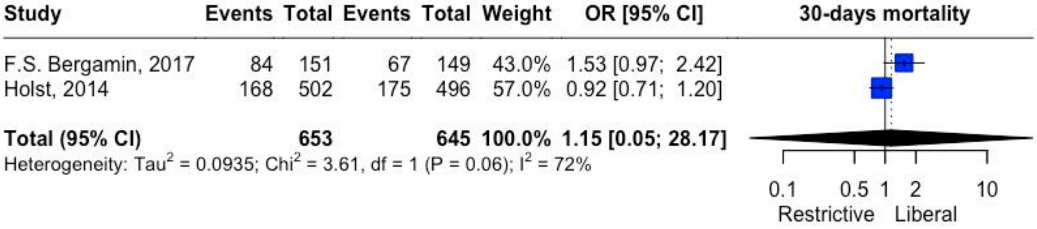

Figure S4. Subgroup analysis 30-days mortality. A. Cardiovascular patients. B. Septic shock patients. C. Medical ICU patients. ICU: Intensive care unit.

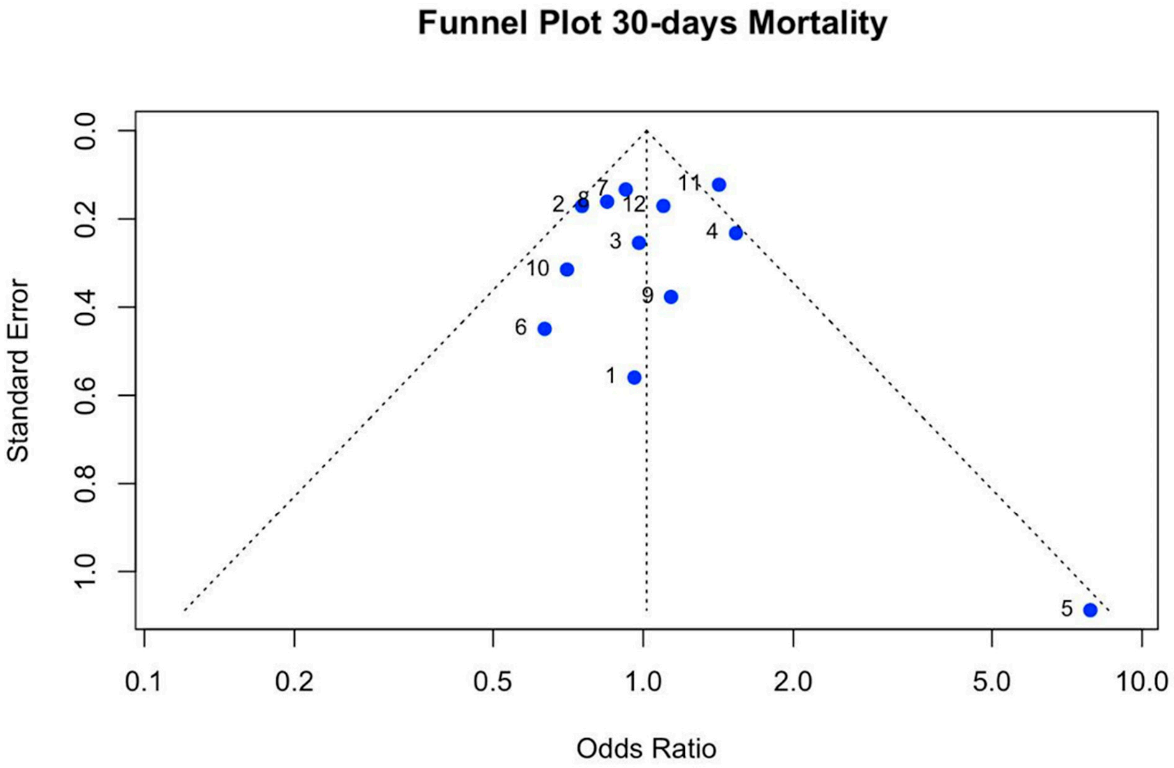

Figure S5. Funnel Plot for 30-days mortality. 1: Hébert, et al 1995, 2: Hébert, et al 1999, 3: Hebert, et al 2001 ; 4 : Bergamin, et al 2017, 5 : Carson, et al 2013 ; 6 : Walsh, et al 2013 ; 7 : Holst, et al 2014 ; 8 : Mazer, et al 2017 ; 9 : Palmieri, et al 2017 ; 10 : Ducrocq, et al 2021 ; 11 : Carson, et al 2020 ; 12 : Taccone, et al 2024.
